# Binge Eating Disorder in Patients with Bipolar Disorder and Relationship with Clinical Features

**DOI:** 10.1192/j.eurpsy.2022.428

**Published:** 2022-09-01

**Authors:** B. Karabiyik, O. Sahmelikoglu Onur, N. Karamustafalioglu

**Affiliations:** Bakirkoy Research & Training Hospital for Psychiatry, Neurology and Neurosurgery, Psychiatry, Istanbul, Turkey

**Keywords:** obesity, eating disorder, bipolar disorder, binge eating disorder

## Abstract

**Introduction:**

Current studies indicate a strong relationship between Eating Disorders and obesity, while studies on Bipolar Disorder (BPD) show that patients with BPD form an important risk group in terms of obesity.

**Objectives:**

The aim of this study is to investigate the frequency of Binge Eating Disorder (BED) in patients diagnosed with euthymic Bipolar Disorder 1 (BPD 1), and the relationship between their clinical features

**Methods:**

This study included 150 patients between 18-65 years of age, diagnosed with euthymic BPD 1 according to DSM 5 criteria. Structured Clinical Interview for DSM-5 Disorders, Structured Sociodemographic Form, Young Mania Scale, Beck Depression Scale, Eating Disorders Assessment Scale (EDAS) , Eating Attitude Test (EAT) were applied to participants.

**Results:**

A diagnosis of BED was detected in 19.3% of the patients. Body weight, highest weight and BMI values were significantly higher in those who were diagnosed with BED compared to those who were not diagnosed with BED. Most of the diagnosed with BED are women; gender was found to be determinant for BED. The total and subscale scores of EAT and EDAS of those with a diagnosis of BED were statistically significantly higher than those who did not. The rate of attacks with psychotic symptoms, rapid cycling and presence of suicide attempt were significantly higher in those with a diagnosis of BED compared to those who did not.

**Conclusions:**

BED may be frequent in BPD 1 patients. Noticing BED in BPD1 patients might help both the more effective treatment of BPD and the prevention of obesity.

**Disclosure:**

No significant relationships.

